# Supplementary material for: Association between On-Treatment Haemoglobin A1c and All-Cause Mortality in Individuals with Type 2 Diabetes: Importance of Personalized Goals and Type of Anti-Hyperglycaemic Treatment
Source: J Clin Med. 2020 Jan 17;9(1):246. doi: 10.3390/jcm9010246 (PMC7019548; doi:10.3390/jcm9010246)
Supplement: Supplementary file 1 [file jcm-09-00246-s001.pdf]

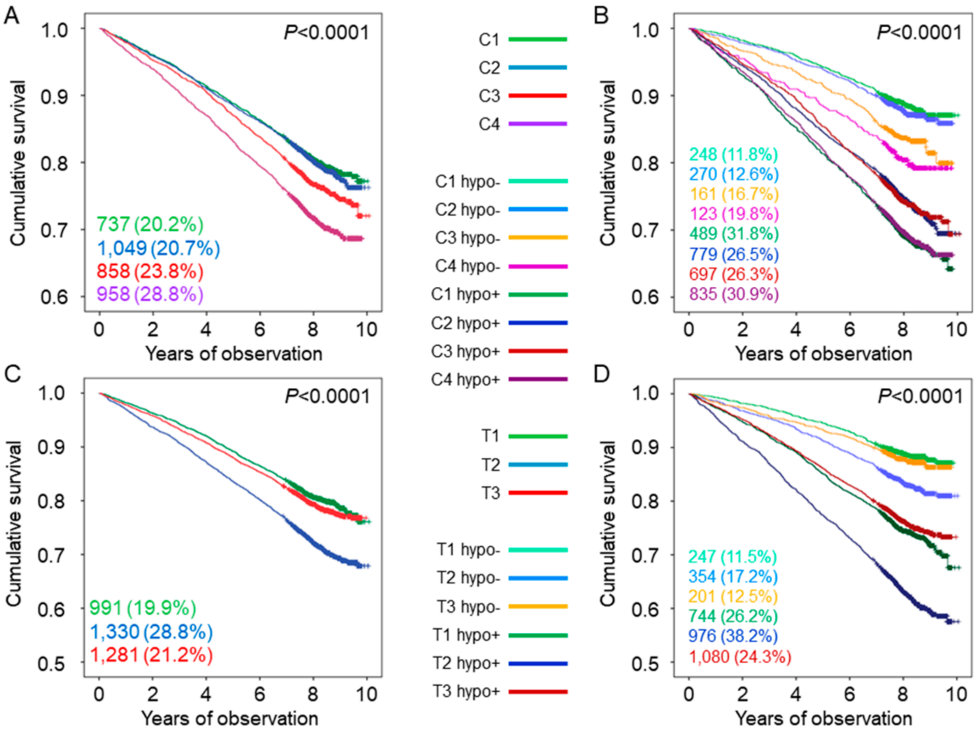

Figure S1. Kaplan-Meier survival curves for all-cause mortality by HbA_{1c} and HbA_{1c} target categories. Cumulative survival by Kaplan-Meier analysis by HbA_{1c} categories (A), HbA_{1c} categories plus use of agents causing hypoglycaemia (B), HbA_{1c} target categories (C), and HbA_{1c} target categories plus use of agents causing hypoglycaemia (D). Number (percentage) of deaths or HRs (95% CI) for mortality are shown for each category. HbA_{1c} = haemoglobin A_{1c}; HR = hazard ratio; CI = confidence interval; C1 = HbA_{1c} <6.5%; C2 = HbA_{1c} 6.5%-7.49%; C3 = HbA_{1c} 7.5%-8.49%; C4 = HbA_{1c} ≥8.5%; T1 = HbA_{1c} on-target (≤0.5% below or above personalized goal); T2 = HbA_{1c} below-target (>0.5% below personalized goal); T3 = HbA_{1c} above-target (>0.5% above personalized goal); hypo- = patients not on agents causing hypoglycaemia; hypo+ = patients on agents causing hypoglycaemia.

Table S1. Distribution of study participants according to personalized HbA_{1c} goals and target categories.

Personalized HbA_{1c} goals	Target categories			
	**All**	**T1**	**T2**	**T3**
**All**				
**G0**	3,859 (24.6)	1,401 (28.1)	674 (14.6)	1,784 (29.5)
**G1**	4,776 (30.5)	1,557 (31.1)	1,194 (25.8)	2,025 (33.5)
**G2**	4,508 (28.8)	1,392 (27.9)	1,553 (33.6)	1,563 (25.9)
**G3**	2,128 (13.6)	548 (11.0)	969 (21.0)	611 (10.1)
**G4**	385 (2.5)	91 (1.8)	231 (5.0)	63 (1.2)
**Total**	15,656	4,989 (31.9)	4,621 (29.5)	6,046 (38.6)
**Hypo-**				
**G0**	2,331 (40.0)	1,019 (47.3)	526 (25.5)	786 (48.9)
**G1**	1,871 (32.1)	666 (30.9)	709 (34.4)	496 (30.8)
**G2**	1,184 (20.3)	369 (17.1)	574 (27.8)	241 (15.0)
**G3**	383 (6.6)	93 (4.3)	213 (10.3)	77 (4.8)
**G4**	57 (1.0)	7 (0.3)	42 (2.0)	8 (0.5)
**Total**	5,826	2,154 (37.0)	2,064 (35.4)	1,608 (27.6)
**Hypo+**				
**G0**	1,528 (15.5)	382 (13.5)	148 (5.8)	998 (22.5)
**G1**	2,905 (29.6)	891 (31.4)	485 (19.0)	1,529 (34.5)
**G2**	3,324 (33.8)	1,023 (36.1)	979 (38.3)	1,322 (29.8)
**G3**	1,745 (17.8)	455 (16.0)	756 (29.6)	534 (12.0)
**G4**	328 (3.3)	84 (3.0)	189 (7.4)	55 (1.2)
**Total**	9,830	2,835 (28.8)	2,557 (26.0)	4,438 (45.2)

HbA_{1c} = haemoglobin A_{1c}; G0 = personalized HbA_{1c} goal <6.5%; G1 = personalized HbA_{1c} goal <7.0%; G2 = personalized HbA_{1c} goal <7.5%; G3 = personalized HbA_{1c} goal <8.0%; G4 = personalized HbA_{1c} goal <8.5%; T1 = HbA_{1c} on-target ($\leq$0.5% below or above personalized goal); T2 = HbA_{1c} below-target (>0.5% below personalized goal); T3 = HbA_{1c} above-target (>0.5% above personalized goal); hypo- = patients not on agents causing hypoglycaemia; hypo+ = patients on agents causing hypoglycaemia.

Table S2. Baseline clinical features of study participants by HbA_{1c} categories plus use of agents causing hypoglycaemia.

Variables	Hypo-				Hypo+				*P*
	C1	C2	C3	C4	C1	C2	C3	C4	
N (%)	2,107 (13.5)	2,137 (13.6)	962 (6.1)	620 (4.0)	1,538 (9.8)	2,944 (18.8)	2,646 (16.9)	2,702 (17.3)	
Deaths, n (%)	248 (11.8%)	270 (12.6%)	161 (16.7%)	123 (19.8%)	489 (31.8%)	779 (26.5%)	697 (26.3%)	835 (30.9%)	<0.001
Age, years	63.5±10.1	65.0±9.8	64.9±10.3	64.2±11.4	68.6±10.4	68.6±9.7	68.0±10.0	66.8±10.6	<0.001
Male gender, n (%)	1,256 (59.6)	1,193 (55.8)	547 (56.9)	338 (54.5)	977 (63.5)	1,710 (58.1)	1,471 (55.6)	1,410 (52.2)	<0.001
Smoking, n (%)									0.01
Never	1,175 (55.8)	1,204 (56.3)	552 (57.4)	340 (54.8)	846 (55.0)	1,646 (55.9)	1,538 (58.1)	1,548 (57.3)	
Former	631 (29.9)	606 (28.4)	269 (28.0)	166 (26.8)	476 (30.9)	866 (29.4)	691 (26.1)	702 (26.0)	
Current	301 (14.3)	327 (15.3)	141 (14.7)	114 (18.4)	216 (14.0)	432 (14.7)	417 (15.8)	452 (16.7)	
Diabetes duration, years	6.5±7.2	9.2±9.0	12.8±9.8	12.9±11.3	12.5±9.8	14.8±9.9	16.8±10.0	16.8±9.8	<0.001
HbA_{1c}, %	5.93±0.44	6.93±0.28	7.89±0.27	9.85±1.49	5.94±0.48	7.01±0.28	7.95±0.29	9.76±1.26	<0.001
($mmol \cdot mol^{-1}$)	(41.3±4.8)	(52.2±3.1)	(62.7±3.0)	(84.2±16.3)	(41.4±5.2)	(53.1±3.1)	(63.4±3.2)	(83.2±13.8)	
BMI, $kg \cdot m^{-2}$	29.1±5.0	29.3±5.0	29.7±5.2	30.2±5.5	27.8±4.7	28.2±4.8	28.8±5.2	29.6±5.5	<0.001
Waist circumference, cm	102.9±10.1	103.2±10.1	104.1±10.6	105.0±11.3	100.3±9.7	100.9±9.7	102.2±10.6	103.8±11.0	<0.001
Triglycerides, $mmol \cdot l^{-1}$	1.48±0.94	1.53±0.89	1.61±0.91	1.93±1.43	1.42±0.95	1.45±0.82	1.54±0.88	1.83±1.24	<0.001
Total cholesterol, $mmol \cdot l^{-1}$	4.85±0.95	4.86±0.96	4.80±0.93	5.01±1.14	4.59±0.96	4.70±0.95	4.73±0.97	4.86±1.08	<0.001
HDL cholesterol, $mmol \cdot l^{-1}$	1.32±0.35	1.30±0.33	1.26±0.31	1.20±0.32	1.30±0.37	1.32±0.37	1.29±0.36	1.25±0.36	<0.001
LDL cholesterol, $mmol \cdot l^{-1}$	3.53±0.92	3.57±0.92	3.54±0.91	3.80±1.10	3.29±0.91	3.38±0.90	3.44±0.92	3.61±1.03	<0.001
Non-HDL cholesterol, $mmol \cdot l^{-1}$	2.87±0.84	2.87±0.83	2.82±0.81	2.95±0.90	2.66±0.83	2.73±0.82	2.74±0.83	2.80±0.90	<0.001
Systolic BP, mmHg	135.5±16.6	136.6±16.8	139.6±18.0	137.9±17.6	137.3±18.7	138.8±18.4	139.6±18.2	138.8±18.9	<0.001
Diastolic BP, mmHg	79.6±9.2	79.5±8.9	80.6±9.4	79.9±9.0	78.2±9.6	77.9±9.4	78.2±9.5	78.4±9.8	<0.001
Pulse pressure, mmHg	55.9±14.3	57.1±14.8	58.9±15.0	57.9±14.9	59.1±16.3	60.9±16.0	61.4±16.2	60.4±15.9	<0.001
Anti-hyperglycaemic treatment, n (%)									
Lifestyle	1,017 (48.3)	762 (35.7)	192 (20.0)	142 (22.9)	0 (0.0)	0 (0.0)	0 (0.0)	0 (0.0)	<0.001
Insulin	0 (0.0)	0 (0.0)	0 (0.0)	0 (0.0)	440 (28.6)	913 (31.0)	1,062 (40.1)	1,509 (55.8)	<0.001
Non-insulin agents	1,090 (51.7)	1,375 (64.3)	770 (80.0)	478 (77.1)	1,098 (71.4)	2,031 (69.0)	1,584 (59.9)	1,193 (44.2)	<0.001
Metformin	1,063 (50.5)	1,346 (63.0)	746 (77.5)	447 (72.1)	683 (44.4)	1,494 (50.7)	1,460 (55.2)	1,406 (52.0)	<0.001
Pioglitazone	58 (2.8)	84 (3.9)	67 (7.0)	53 (8.5)	29 (1.9)	83 (2.8)	96 (3.6)	84 (3.1)	<0.001
Acarbose	15 (0.7)	16 (0.7)	21 (2.2)	20 (3.2)	21 (1.4)	27 (0.9)	26 (1.0)	24 (0.9)	<0.001
Sulfonylureas	0 (0.0)	0 (0.0)	0 (0.0)	0 (0.0)	820 (53.3)	1,677 (57.0)	1,472 (55.6)	1,281 (47.4)	<0.001
Repaglinide	0 (0.0)	0 (0.0)	0 (0.0)	0 (0.0)	335 (21.8)	537 (18.2)	371 (14.0)	282 (10.4)	<0.001
Lipid-lowering treatment, n (%)	901 (42.8)	1,007 (47.1)	419 (43.6)	263 (42.4)	671 (43.6)	1,382 (46.9)	1,309 (49.5)	1,286 (47.6)	<0.001
Anti-hypertensive treatment, n (%)	1,396 (66.3)	1,458 (68.2)	664 (69.0)	386 (62.3)	1,113 (72.4)	2,153 (73.1)	1,937 (73.2)	1,965 (72.7)	<0.001
Albuminuria, $mg \cdot day^{-1}$	33.8±148.1	46.8±392.8	41.6±165.7	52.3±217.5	81.1±298.6	84.7±357.4	86.4±371.1	106.0±307.0	<0.001
Serum creatinine, $\mu mol \cdot l^{-1}$	76.0±23.9	76.0±30.9	76.0±20.3	75.1±23.9	89.3±50.4	84.0±37.1	82.2±35.4	83.1±33.6	<0.001
eGFR, $ml \cdot min^{-1} \cdot 1.73m^{-2}$	85.6±18.6	84.3±17.8	84.1±18.0	85.2±20.5	76.1±22.8	77.3±21.1	78.3±21.2	77.8±22.8	<0.001
DKD phenotype, n (%)									<0.001
No DKD	1,649 (78.3)	1,600 (74.9)	679 (70.6)	391 (63.1)	905 (58.8)	1,785 (60.6)	1,590 (60.1)	1,385 (51.3)	
Albuminuric DKD with preserved eGFR	260 (12.3)	323 (15.1)	177 (18.4)	156 (25.2)	272 (17.7)	554 (18.8)	514 (19.4)	710 (26.3)	
Non-albuminuric DKD	127 (6.0)	149 (7.0)	73 (7.6)	43 (6.9)	194 (12.6)	316 (10.7)	287 (10.8)	287 (10.6)	
Albuminuric DKD with reduced eGFR	71 (3.4)	65 (3.0)	33 (3.4)	30 (4.8)	167 (10.9)	289 (9.8)	255 (9.6)	320 (11.8)	
DR, n (%)									<0.001
No DR	1,980 (94.0)	1,967 (92.0)	817 (84.9)	491 (79.2)	1,198 (77.9)	2,218 (75.3)	1,841 (69.6)	1,677 (62.1)	
Non-advanced DR	80 (3.8)	115 (5.4)	109 (11.3)	90 (14.5)	162 (10.5)	388 (13.2)	477 (18.0)	526 (19.5)	
Advanced DR	47 (2.2)	55 (2.6)	36 (3.7)	39 (6.3)	178 (11.6)	338 (11.5)	328 (12.4)	499 (18.5)	

**CVD, n (%)**									
**Any**	266 (12.6)	363 (17.0)	202 (21.0)	153 (24.7)	433 (28.2)	723 (24.6)	704 (26.6)	776 (28.7)	<0.001
**Myocardial infarction**	138 (6.5)	156 (7.3)	83 (8.6)	63 (10.2)	216 (14.0)	358 (12.2)	362 (13.7)	366 (13.5)	<0.001
**Coronary revascularization**	113 (5.4)	162 (7.6)	97 (10.1)	59 (9.5)	182 (11.8)	325 (11.0)	314 (11.9)	327 (12.1)	<0.001
**Stroke**	45 (2.1)	41 (1.9)	25 (2.6)	11 (1.8)	81 (5.3)	102 (3.5)	93 (3.5)	115 (4.3)	<0.001
**Carotid revascularization**	38 (1.8)	105 (4.9)	74 (7.7)	72 (11.6)	82 (5.3)	135 (4.6)	161 (6.1)	189 (7.0)	<0.001
**Ulcer/gangrene/amputation**	21 (1.0)	31 (1.5)	15 (1.6)	15 (2.4)	71 (4.6)	136 (4.6)	116 (4.4)	151 (5.6)	<0.001
**Lower limb revascularization**	18 (0.9)	38 (1.8)	29 (3.0)	26 (4.2)	47 (3.1)	97 (3.3)	92 (3.5)	103 (3.8)	<0.001
**Comorbidities n (%)**									
**Any**	311 (14.8)	325 (15.2)	142 (14.8)	101 (16.3)	371 (24.1)	547 (18.6)	478 (18.1)	512 (18.9)	<0.001
**COPD**	96 (4.6)	56 (2.6)	29 (3.0)	17 (2.7)	77 (5.0)	145 (4.9)	113 (4.3)	141 (5.2)	<0.001
**Chronic liver disease**	120 (5.7)	177 (8.3)	86 (8.9)	69 (11.1)	179 (11.6)	238 (8.1)	240 (9.1)	252 (9.3)	<0.001
**Cancer**	124 (5.9)	121 (5.7)	36 (3.7)	22 (3.5)	155 (10.1)	221 (7.5)	179 (6.8)	173 (6.4)	<0.001

HbA_{1c} = haemoglobin A_{1c}; hypo- = patients not on agents causing hypoglycaemia; hypo+ = patients on agents causing hypoglycaemia; C1 = HbA_{1c} <6.5%; C2 = HbA_{1c} 6.5%-7.49%; C3 = HbA_{1c} 7.5%-8.49%; C4 = HbA_{1c} ≥8.5%; BMI = body mass index; BP = blood pressure; ACE-I = angiotensin converting enzyme inhibitor; ARB = angiotensin receptor blocker; eGFR = estimated glomerular filtration rate; DKD = diabetic kidney disease; DR = diabetic retinopathy; = CVD = cardiovascular disease; COPD = chronic obstructive pulmonary disease.

Table S3. Baseline clinical features of study participants by HbA_{1c} target categories and use of agents causing hypoglycaemia.

Variables	Hypo-			Hypo+			*P*
	T1	T2	T3	T1	T2	T3	
**N (%)**	2,154 (11.5)	2,064 (17.2)	1,608 (12.5)	2,835 (26.2)	2,557 (38.2)	4,438 (24.3)	
**Deaths, n (%)**	247 (11.5%)	354 (17.2%)	201 (12.5%)	744 (26.2%)	976 (38.2%)	1,080 (24.3%)	<0.001
**Age, years**	64.0±9.6	66.5±10.4	61.9±10.2	68.7±9.7	71.7±9.1	65.2±10.2	<0.001
**Male gender, n (%)**	1,247 (57.9)	1,201 (58.2)	886 (55.1)	1,586 (55.9)	1,617 (63.2)	2,365 (53.3)	<0.001
**Smoking, n (%)**							<0.001
**Never**	1,204 (55.9)	1,179 (57.1)	888 (55.2)	1,633 (57.6)	1,402 (54.8)	2,543 (57.3)	
**Former**	645 (29.9)	621 (30.1)	406 (25.2)	790 (27.9)	834 (32.6)	1,111 (25.0)	
**Current**	305 (14.2)	264 (12.8)	314 (19.5)	412 (14.5)	321 (12.6)	784 (17.7)	
**Diabetes duration, years**	8.5±8.5	9.5±9.6	9.8±9.4	15.8±10.1	16.3±10.5	14.9±9.6	<0.001
**HbA_{1c}, %**	6.87±0.51	6.04±0.59	8.53±1.43	7.34±0.54	6.37±0.67	9.03±1.36	<0.001
**(mmol·mol^{-1})**	(51.6±5.6)	(42.5±6.4)	(69.7±15.6)	(56.7±5.9)	(46.1±7.3)	(75.2±14.9)	
**BMI, kg·m^{-2}**	29.4±5.0	28.8±4.9	30.2±5.5	28.3±4.9	27.8±4.6	29.5±5.5	<0.001
**Waist circumference, cm**	103.5±10.1	102.2±9.9	105.0±11.1	101.1±9.9	100.2±9.4	103.4±11.0	<0.001
**Triglycerides, mmol·l^{-1}**	1.55±0.98	1.43±0.83	1.76±1.16	1.48±0.82	1.41±0.88	1.73±1.14	<0.001
**Total cholesterol, mmol·l^{-1}**	4.88±0.93	4.79±0.95	4.94±1.05	4.70±0.95	4.61±0.96	4.84±1.03	<0.001
**HDL cholesterol, mmol·l^{-1}**	1.29±0.33	1.33±0.36	1.23±0.32	1.31±0.36	1.32±0.37	1.26±0.36	<0.001
**LDL cholesterol, mmol·l^{-1}**	3.58±0.91	3.46±0.91	3.71±1.02	3.39±0.89	3.29±0.92	3.57±0.99	<0.001
**Non-HDL cholesterol, mmol·l^{-1}**	2.89±0.82	2.81±0.82	2.92±0.88	2.72±0.82	2.66±0.83	2.79±0.87	<0.001
**Systolic BP, mmHg**	136.6±16.7	136.7±17.0	137.3±17.6	139.7±18.6	138.3±18.6	138.6±18.4	<0.001
**Diastolic BP, mmHg**	79.8±8.8	79.4±9.2	80.3±9.3	78.4±9.4	77.5±9.7	78.4±9.6	<0.001
**Pulse pressure, mmHg**	56.8±14.6	57.3±14.7	57.0±14.8	61.3±16.1	60.8±16.4	60.1±15.9	<0.001
**Anti-hyperglycaemic treatment, n (%)**							
**Lifestyle**	810 (37.6)	927 (44.9)	376 (23.4)	0 (0.0)	0 (0.0)	0 (0.0)	<0.001
**Insulin**	0 (0.0)	0 (0.0)	0 (0.0)	972 (33.6)	860 (34.3)	2,092 (47.1)	<0.001
**Non-insulin agents**	1,344 (62.4)	1,137 (55.1)	1,232 (76.6)	1,863 (65.7)	1,697 (66.4)	2,346 (52.9)	<0.001
**Metformin**	1,310 (60.8)	1,111 (53.8)	1,181 (73.4)	1,474 (52.0)	1,084 (42.4)	2,485 (56.0)	<0.001
**Pioglitazone**	89 (4.1)	52 (2.5)	121 (7.5)	92 (3.2)	40 (1.6)	160 (3.6)	<0.001
**Acarbose**	17 (0.8)	21 (1.0)	34 (2.1)	32 (1.1)	26 (1.0)	40 (0.9)	<0.001
**Sulfonylureas**	0 (0.0)	0 (0.0)	0 (0.0)	1,610 (56.8)	1,314 (51.4)	2,326 (52.4)	<0.001
**Repaglinide**	0 (0.0)	0 (0.0)	0 (0.0)	461 (16.3)	510 (19.9)	554 (12.5)	<0.001
**Lipid-lowering treatment, n (%)**	948 (44.0)	972 (47.1)	670 (41.7)	1,366 (48.2)	1,176 (46.0)	2,106 (47.5)	<0.001
**Anti-hypertensive treatment, n (%)**	1,417 (65.8)	1,475 (71.5)	1,012(62.9)	2,087 (73.6)	1,974 (77.2)	3,107 (70.0)	<0.001
**Albuminuria, mg·day^{-1}**	31.8±114.0	53.8±413.8	39.9±176.4	88.7±363.5	101.7±343.1	85.0±320.9	<0.001
**Serum creatinine, µmol·l^{-1}**	75.1±29.2	77.8±26.5	74.3±21.2	83.1±33.6	92.8±50.4	80.4±30.9	<0.001
**eGFR, ml·min^{-1}·1.73m^{-2}**	85.5±17.0	82.3±19.3	87.1±18.6	77.5±20.9	71.9±22.3	80.8±21.6	<0.001
**DKD phenotype, n (%)**							<0.001
**No DKD**	1,656 (76.9)	1,520 (73.6)	1,143 (71.1)	1,692 (59.7)	1,339 (52.4)	2,634 (59.4)	
**Albuminuric DKD with preserved eGFR**	312 (14.5)	284 (13.8)	320 (19.9)	551 (19.4)	483 (18.9)	1,016 (22.9)	
**Non-albuminuric DKD**	140 (6.5)	161 (7.8)	91 (5.7)	321 (11.3)	362 (14.2)	401 (9.0)	

**Albuminuric DKD with reduced eGFR**	46 (2.1)	99 (4.8)	54 (3.4)	271 (9.6)	373 (14.6)	387 (8.7)	
**DR, n (%)**							<0.001
**No DR**	2,000 (92.9)	1,870 (90.6)	1,385 (86.1)	2,064 (72.8)	1,825 (71.4)	3,045 (68.6)	
**Non-advanced DR**	117 (5.4)	109 (5.3)	168 (10.4)	412 (14.5)	335 (13.1)	806 (18.2)	
**Advanced DR**	37 (1.7)	85 (4.1)	55 (3.4)	359 (12.7)	397 (15.5)	587 (13.2)	
**CVD, n (%)**							
**Any**	291 (13.5)	478 (23.2)	215 (13.4)	713 (25.1)	979 (38.3)	944 (21.3)	<0.001
**Myocardial infarction**	125 (5.8)	221 (10.7)	94 (5.8)	355 (12.5)	482 (18.9)	465 (10.5)	<0.001
**Coronary revascularization**	134 (6.2)	208 (10.1)	89 (5.5)	313 (11.0)	409 (16.0)	426 (9.6)	<0.001
**Stroke**	35 (1.6)	67 (3.2)	20 (1.2)	102 (3.6)	164 (6.4)	125 (2.8)	<0.001
**Carotid revascularization**	88 (4.1)	114 (5.5)	87 (5.4)	162 (5.7)	194 (7.6)	211 (4.8)	<0.001
**Ulcer/gangrene/amputation**	23 (1.1)	41 (2.0)	18 (1.1)	135 (4.8)	176 (6.9)	163 (3.7)	<0.001
**Lower limb revascularization**	33 (1.5)	46 (2.2)	32 (2.0)	89 (3.1)	125 (4.9)	125 (2.8)	<0.001
**Comorbidities n (%)**							
**Any**	239 (11.1)	479 (23.2)	161 (10.0)	517 (18.2)	782 (30.6)	609 (13.7)	<0.001
**COPD**	41 (1.9)	132 (6.4)	25 (1.6)	126 (4.4)	190 (7.4)	160 (3.6)	<0.001
**Chronic liver disease**	135 (6.3)	212 (10.3)	105 (6.5)	250 (8.8)	358 (14.0)	301 (6.8)	<0.001
**Cancer**	75 (3.5)	187 (9.1)	41 (2.5)	201 (7.1)	317 (12.4)	210 (4.7)	<0.001

HbA_{1c} = haemoglobin A_{1c}; hypo- = patients not on agents causing hypoglycaemia; hypo+ = patients on agents causing hypoglycaemia; T1 = HbA_{1c} on-target (≤0.5% below or above personalized goal); T2 = HbA_{1c} below-target (>0.5% below personalized goal); T3 = HbA_{1c} above-target (>0.5% above personalized goal); BMI = body mass index; BP = blood pressure; ACE-I = angiotensin converting enzyme inhibitor; ARB = angiotensin receptor blocker; eGFR = estimated glomerular filtration rate; DKD = diabetic kidney disease; DR = diabetic retinopathy; = CVD = cardiovascular disease; COPD = chronic obstructive pulmonary disease.

The RIACE Study Group

The RIACE Steering Committee

Giuseppe Pugliese (Coordinator), Giuseppe Penno (Secretary), Anna Solini, Enzo Bonora, Emanuela Orsi, Roberto Trevisan, Luigi Laviola, Antonio Nicolucci.

Participating Diabetes Centres

1. Azienda Ospedaliera Sant'Andrea, Roma (Coordinating Centre): Giuseppe Pugliese, Lucilla Bollanti, Elena Alessi, Martina Vitale, and Tiziana Cirrito.
2. Ospedale Le Molinette, Torino: Paolo Cavallo-Perin, Gabriella Gruden, and Bartolomeo Lorenzati.
3. Ospedale San Luigi Gonzaga, Orbassano: Franco Cavalot, Mariella Trovati, Leonardo Di Martino, and Fabio Mazzaglia.
4. Ospedale San Raffaele, Milano: Giampaolo Zerbini, Valentina Martina, Silvia Maestroni, and Valentina Capuano.
5. IRCCS "Cà Granda – Ospedale Maggiore Policlinico", Milano: Emanuela Orsi, Eva Palmieri, Elena Lunati, Valeria Grancini, and Veronica Resi.
6. Ospedale San Paolo, Milano: Antonio Pontiroli, Annamaria Veronelli, and Barbara Zecchini.
7. Ospedale San Giuseppe, Milano: Maura Arosio, Laura Montefusco, Antonio Rossi, and Guido Adda.
8. ASST - Ospedale Papa Giovanni XXIII, Bergamo: Roberto Trevisan, Anna Corsi, and Mascia Albizzi.
9. Ospedale Maggiore, Verona: Enzo Bonora, and Giacomo Zoppini.
10. Policlinico Universitario, Padova: Angelo Avogaro, and Monica Vedovato.
11. Ospedale Cisanello, Azienda Ospedaliero-Universitaria Pisana, Pisa: Giuseppe Penno, Laura Pucci, Daniela Lucchesi, Eleonora Russo, and Monia Garofolo.
12. Ospedale Santa Chiara, Azienda Ospedaliero-Universitaria Pisana, Pisa: Anna Solini.
13. Ospedale Le Scotte, Siena: Francesco Dotta, Cecilia Fondelli, and Laura Nigi.
14. Policlinico Umberto I, Roma: Susanna Morano, Tiziana Filardi, Irene Turinese, and Marco Rossetti.
15. Ospedale S. Maria Goretti, Latina: Raffaella Buzzetti and Chiara Foffi.
16. Ospedali Riuniti, Foggia: Mauro Cignarelli, Olga Lamacchia, Sabina Pinnelli, and Lucia Monaco.
17. Policlinico Universitario, Bari: Francesco Giorgino, Luigi Laviola, and Annalisa Natalicchio.
18. Policlinico Mater Domini, Catanzaro: Giorgio Sesti and Francesco Andreozzi.
19. Policlinico Monserrato, Cagliari: Marco Giorgio Baroni, Giuseppina Frau, and Alessandra Boi.
